# A Novel LC-APCI-MS/MS Approach for the Trace Analysis of 3,4-Difluoronitrobenzene in Linezolid

**DOI:** 10.3390/ph18040465

**Published:** 2025-03-26

**Authors:** Yujin Lim, Aelim Kim, Eunyeong Shin, Hwangeui Cho

**Affiliations:** Institute of New Drug Development, School of Pharmacy, Jeonbuk National University, Jeonju 54896, Republic of Korea

**Keywords:** 3,4-difluoronitrobenzene (DFNB), linezolid, LC-MS/MS, APCI, substitution

## Abstract

**Background/Objectives**: Oxazolidinones are novel antimicrobial agents used to combat bacterial infections, particularly multidrug-resistant strains. However, the synthesis of oxazolidinone derivatives, such as linezolid, often involves the use of 3,4-difluoronitrobenzene (DFNB) as an initiator. Despite its effectiveness, residual DFNB in drug products raises significant health concerns due to its structural similarity to toxic and carcinogenic nitrobenzenes. This contamination is particularly concerning in pharmaceutical formulations, where it poses potential patient safety hazards. Therefore, strict concentration limits for this impurity are necessary. **Methods**: To ensure tight control of DFNB concentrations, this study established an 8.3 µg/g target limit. An advanced high-performance liquid chromatography-tandem mass spectrometry (LC-MS/MS) method was developed to overcome current limitations in detecting trace DFNB. Under negative atmospheric pressure chemical ionization (APCI) conditions, DFNB exhibited characteristic ion formations, including [M]^•−^ through electron capture and [M − F + O]^−^ via substitution reactions. The quantitative method utilizes MS/MS ion transitions of the substitution product while optimizing chromatographic and spectrometric parameters to enhance both sensitivity and specificity. **Conclusions**: Validation tests confirm the efficiency, precision, and accuracy of this method, with a low limit of quantification (LOQ) of 5 ng/mL (0.83 µg/g). This technique enables accurate detection and quantification of DFNB in linezolid active pharmaceutical ingredient (API) and various formulations, providing a reliable tool for quality control. This method ensures the safe use of linezolid by effectively monitoring and minimizing the risks associated with DFNB contamination.

## 1. Introduction

**Figure 1:**
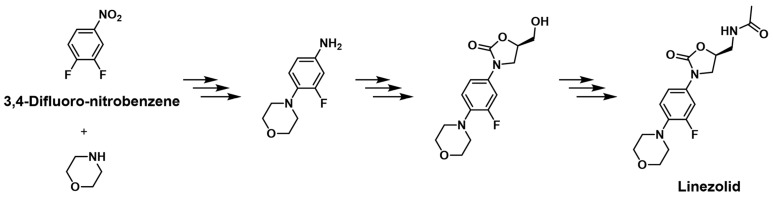
Linezolid synthesis initiated by 3,4-difluoronitrobenzene (stacked arrows indicate the multistep synthesis/reaction).

Oxazolidinones represent a novel class of antimicrobial agents that have shown remarkable effectiveness in treating various bacterial infections, including those caused by multidrug-resistant strains [1,2,3,4]. The synthesis of specific oxazolidinone derivatives, such as linezolid and eperazolid, involves the use of an initiator such as 3,4-difluoronitrobenzene (DFNB), a fluorinated nitroaromatic compound (Figure 1) [5]. Despite its utility in the synthesis process, the presence of 3,4-difluoronitrobenzene as a residual impurity in the final drug product raises concerns regarding its potential health risks. Some of chloronitrobenzenes, such as 1-chloro-2-nitrobenzene, 1-chloro-4-nitrobenzene, 1,4-dichloro-2-nitrobenzene, and 2,4-dichloro-1-nitrobenzene, are well documented for their toxicological and carcinogenic properties, with evidence linking them to adverse health effects, including liver and kidney toxicity, genotoxicity, and carcinogenicity [6,7]. In contrast, experimental data on the carcinogenic properties of DFNB remain limited. Due to the structural similarity between chloronitrobenzenes and 3,4-difluoronitrobenzene, there is a growing concern that DFNB may exhibit similar or even enhanced toxicological effects [8]. The presence of fluorine atoms in the 3,4-positions of the nitrobenzene ring could increase the chemical stability, lipophilicity, and persistence of DFNB, potentially leading to bioaccumulation and prolonged exposure risks [9,10]. Furthermore, the fluorine substitution may enhance the reactivity of compounds, raising the possibility of DNA damage and carcinogenesis [11,12].

Considering these concerns, it is imperative to assess the risk of 3,4-difluoronitrobenzene contamination in linezolid drug formulations, particularly as residual levels of this compound could pose a significant threat to patient safety. In this study, to control this potential mutagenic impurity in linezolid, a concentration limit of 8.3 µg/g was established. This limit is derived from a maximum daily dose of 1200 mg for linezolid and a less-than-lifetime (LTL) acceptable intake (AI) of 10 µg/day for the impurity, as outlined in the ICH M7 guideline for short-term exposure duration of 1 to 10 years [13]. However, there is a notable gap in the development of reliable and efficient analytical methods to detect and quantify DFNB residues in pharmaceutical products. Existing detection techniques for nitroaromatic compounds may not provide the required sensitivity and specificity to accurately measure trace amounts of DFNB in the complex matrix of pharmaceutical formulations [14].

Several analytical methods, such as gas chromatography (GC) [15], high-performance liquid chromatography (HPLC) [16,17,18], capillary electrophoresis (CE) [19], electrochemical detection [20,21], and ultraviolet–visible (UV–vis) spectrometry [22,23], have been proposed for detecting nitrobenzene compounds. Among these, GC or HPLC combined with mass spectrometry (MS) stands out as a promising approach due to its high separation efficiency, superior detection sensitivity, and precise identification [14,24,25].

However, analyzing halogenated nitrobenzene compounds like DFNB presents additional challenges due to their unique physical and chemical properties, such as the high electronegativity of fluorine and the electron-withdrawing nature of the nitro-substitution groups [23,26]. Nitrobenzene molecules, being nonpolar, often have poor ionization efficiency under electrospray ionization (ESI), the most commonly used ionization method.

To overcome these limitations, alternative ionization techniques, such as atmospheric pressure chemical ionization (APCI), have been explored to enhance sensitivity, selectivity, and method reliability for ionizing hydrophobic and nonpolar analytes [27]. Several ionization mechanisms, including proton attachment, proton abstraction, anion adduction, electron capture, and dissociative electron capture, have been proposed for APCI [28,29]. For instance, some halogenated nitrobenzenes can undergo both dissociative and nondissociative electron-capture (EC) ionization in the negative ion mode during APCI, leading to the ion formation such as [M–NO]^−^, [M–NO_2_]^−^ and [M]^•−^ [30]. Additionally, halogenated nitrobenzene compounds can form phenoxide ion substitution products, represented as [M − X + O]^−^, through substitution reactions under negative APCI conditions [31,32]. Despite these advancements, there remains limited evaluation of whether specific ionization combined with LC-MS is suitable for precise analysis in complex pharmaceutical matrices. Furthermore, detailed studies on detecting trace levels of potential genotoxic impurities in drug formulations, such as nitrobenzene compounds, are still lacking.

This study aims to develop an advanced analytical method for detecting and quantifying 3,4-difluoronitrobenzene (DFNB) in linezolid. By enabling accurate measurement of DFNB residues, this method will help the pharmaceutical industry better evaluate the safety of linezolid and mitigate potential health risks associated with this hazardous impurity.

## 2. Results and Discussion

### 2.1. Optimization of the MS Conditions

Aromatic nitro compounds, such as nitrobenzenes, are nonpolar molecules that lack proton-donating or proton-accepting functional groups, which limits their ionization through standard protonation or deprotonation mechanisms. Research indicates that, in APCI negative ionization mode, nitroaromatic compounds can generate negative molecular ions via electron capture or charge exchange. Additionally, they may also form substitution products. These ions are commonly observed in aromatic compounds containing nitro or halogen groups.

To explore potential ionization mechanisms applied in this study, APCI-MS spectra were obtained for various solvent systems, including methanol and acetonitrile. Initially, mass spectra were obtained using (-) APCI within the *m*/*z* range of 100–300 to identify specific ions of DFNB for the analytical characterization of the methods.

**Figure 2:**
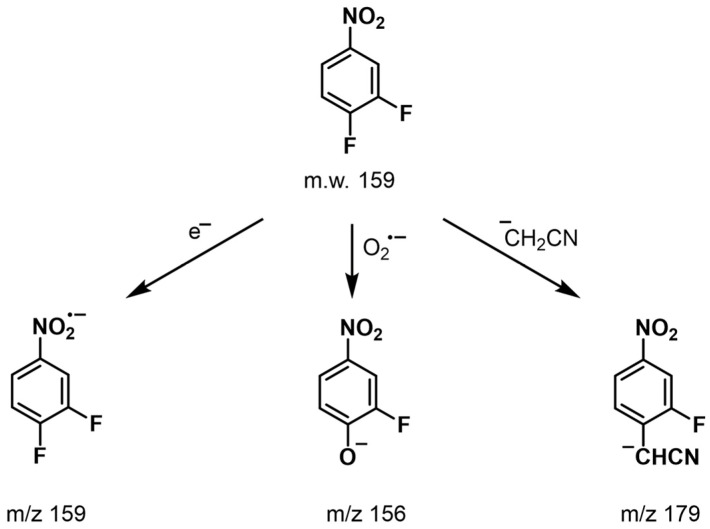
Structure of 3,4-Difluoronitrobenzene (DFNB) and the proposed chemical structure of negative ions formed in the APCI mode.

**Figure 3:**
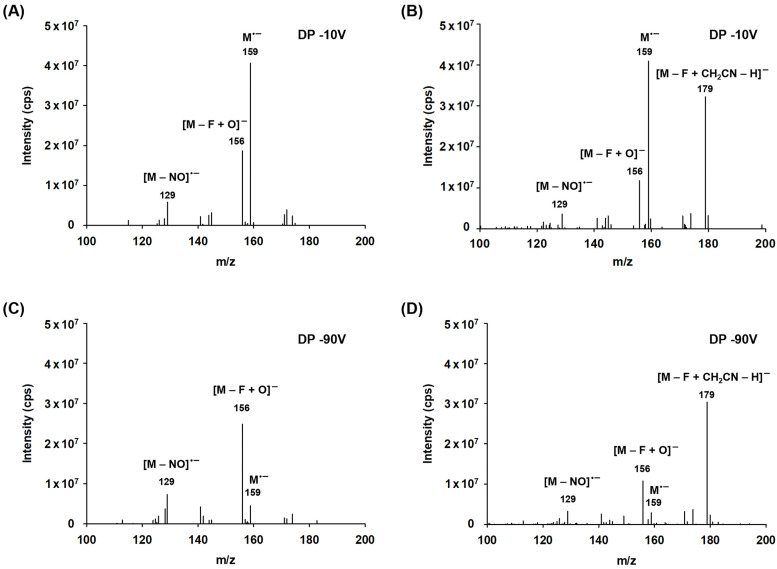
The full-scan spectra obtained for various declustering potentials and solvent systems. (**A**,**C**): methanol as the solvent; (**B**,**D**): acetonitrile as the solvent.

In our study, several distinct regions of anion formation were identified for DFNB (Figure 2). At a lower declustering potential (DP −10 V) with methanol as the solvent, the full-scan spectra exhibit abundant peaks at *m*/*z* 159, corresponding to the negatively charged molecular radical ion [M]^•−^ formed via the electron-capture process, and at *m*/*z* 156, represented as [M − F + O]^−^, indicating the replacement of fluorine with oxygen (Figure 3A). Also, weak signals were observed in the spectrum, including anions formed through the dissociation of the nitro group, such as [M − NO]^−^. At higher declustering potentials (DP −90 V), the spectra were dominated by the phenoxide ion [M − F + O]^−^, accompanied by a weak signal for the [M − NO]^−^ ion, while the intensity of the [M]^•−^ ion decreased significantly (Figure 3C). Herein, the formation of [M − F + O]^−^ likely results from the creation of a superoxide ion (O_2_^•−^) via electron capture, which subsequently reacts chemically with DFNB through the following pathway:

O_2_ + e^−^ → O_2_^•−^

M + O_2_^−^ → [M − F + O]^−^

Such substitution reactions involving halogenated nitroaromatic compounds and O_2_ in APCI have been reported to yield substitution products, even at trace oxygen levels as low as 1 ppm [31]. It is likely that oxygen from the atmosphere or the compressed air used as the nebulizer gas in our mass spectrometry system contributed to these reactions. As an alternative reason, oxygen could have entered the ion source either dissolved in the solvents or from the breakdown of solvent molecules within the ion source [30]. Moreover, substitution is expected to primarily occur at the para position, as the anion in the para-intermediate can be delocalized onto the oxygen atom of the nitro group, significantly increasing the substitution rate compared to the meta position.

**Figure 4:**
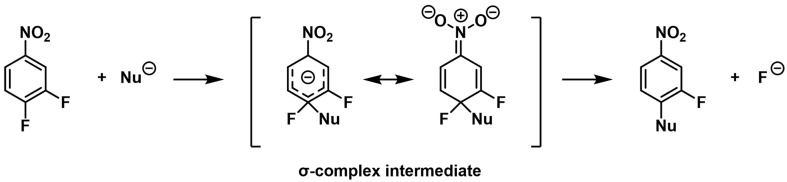
Possible nucleophilic aromatic substitution in 3,4-difluoronitrobenzene (DFNB). Nu: nucleophile.

The solvent played a key role in determining the proportion of substitution products. Both [M]^•−^ and [M − F + O]^−^ were observed with high intensity in experiments using acetonitrile as the solvent (Figure 3B,D). However, the sensitivity of the oxygen-substitution product ion decreased compared to when methanol was used as the solvent. Notably, the 2-fluoro-4-nitrophenylacetonitrile anion [M–F + CH_2_CN–H]^−^ at *m*/*z* 179 was most prominent in the acetonitrile solvent. This anion likely forms via a nucleophilic attack of CH_2_CN^−^, derived from acetonitrile, on DFNB, leading to the formation of an anionic σ-complex (Meisenheimer complex), followed by fluorine elimination and deprotonation (Figure 4) [33,34].

**Figure 5:**
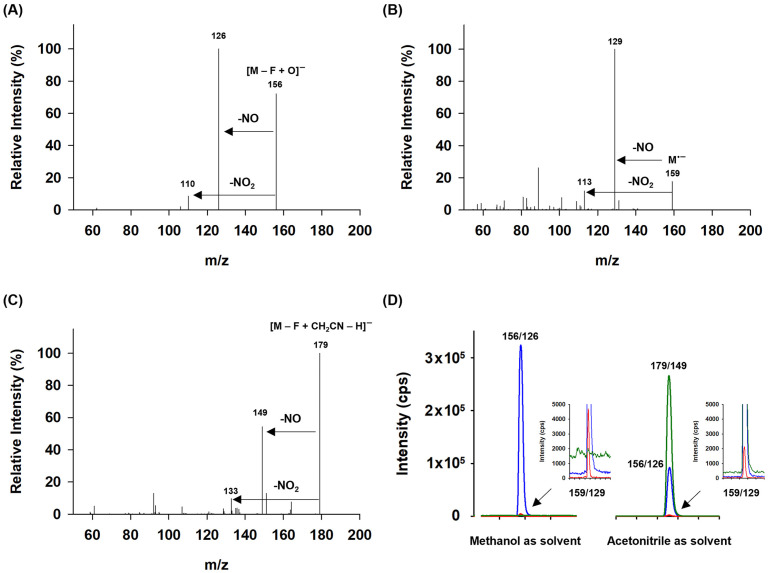
Product ion spectra of DFNB: (**A**) product ion spectrum of [M − F + O]^−^, (**B**) product ion spectrum of [M]^•−^, (**C**) product ion spectrum of [M − F + CH_2_CN − H]^−^, and (**D**) the relative signal responses for the transitions *m*/*z* 156 → 126 (blue), *m*/*z* 159→129 (red), and *m*/*z* 179 → 149 (green).

Tandem mass spectrometry (MS/MS) was conducted in product ion scan mode, utilizing collision-induced dissociation (CID) to produce fragmentation and obtain valuable substructural information (Figure 5). Fragment ions at *m*/*z* 126 and *m*/*z* 110 were attributed to the loss of a nitrosyl radical (NO) and a nitryl radical (NO_2_) from a [M − F + O]^−^ ion at *m*/*z* 156, respectively. Similarly, fragment ions corresponding to the loss of NO and NO_2_ were observed from [M]^•−^ and [M − F + CH_2_CN − H]^−^. However, fragmentation of the molecular radical anion was observed to a limited extent.

This result highlights the feasibility of utilizing oxygen-associated substitution products and their corresponding fragment ions for the selective detection of DFNB. For quantification, the transition of *m*/*z* 156 → 126 was selected due to its high abundance and consistency, while the transition of *m*/*z* 156 → 110 was chosen for identification based on its secondary abundance. Using this approach, further examination has been conducted on the development, optimization, and validation of a specific LC-MS/MS method, in conjunction with chromatography.

### 2.2. Optimization of LC Conditions

To effectively separate the impurity from the complex matrix and minimize peak interference, it was necessary to optimize chromatographic conditions. This process involved systematically adjusting various parameters, including buffer composition, pH, gradient profile, solvent selection, column type, flow rate, column oven temperature, and diluent.

The choice of the mobile phase played a significant role in determining LC–MS sensitivity by influencing analyte retention and ionization. Previous findings indicated that [M − F + O]^−^ formation during DFNB ionization was more prominent when methanol was used as the solvent. Therefore, this study prioritized optimizing chromatographic conditions by selecting appropriate additives and columns for methanol-based mobile phases.

Stationary phases such as C18, F5, and phenyl columns with a particle size of 2.6 μm and a length of 100 mm were evaluated for their suitability in method development. While no significant differences in retention or separation were observed among the reversed-phase columns, the Ascentis^®^ Express C18 column demonstrated the best overall performance in retaining and separating the active pharmaceutical ingredient (API) and impurity.

**Figure 6:**
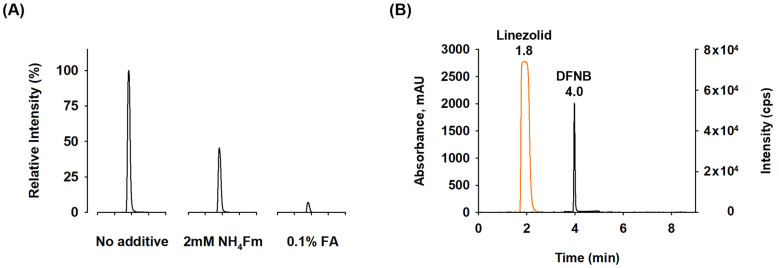
Chromatogram on sensitivity based on mobile phase additives and separation of high concentration API. (**A**) Chromatogram on sensitivity of DFNB according to mobile phase additives, (**B**) LC-UV chromatogram at 252 nm from the injection of a 6 mg/mL linezolid solution and LC-MS chromatogram from the injection of a 50 ng/mL DFNB solution (overlaid).

To further refine the method, volatile buffers and acids were incorporated into the mobile phase to control the ionization state of the analytes and adjust retention. Adding 2 mM ammonium formate buffer or 0.1% formic acid to the mobile phase had minimal impact on the retention of the impurity and the API. In contrast, the use of pure water and methanol as mobile phases without additives resulted in significantly higher formation of a negatively charged molecular ion, where oxygen replaced fluoride (Figure 6A).

To enhance efficiency and reduce run times, this method was further optimized by employing gradient mode separation at a flow rate of 0.3 mL/min and a column temperature of 40 °C. Chromatographic data confirmed the successful separation of DFNB from the excess API, with the impurity eluting after the tail of the API peak (Figure 6B). The final method effectively separated linezolid and its impurity within 9 min.

### 2.3. Method Validation

#### 2.3.1. System Suitability and Specificity

To evaluate injection-to-injection variability, six consecutive injections of the DFNB impurity standard solution were performed at a concentration of 50 ng/mL (equivalent to DFNB in linezolid at 8.3 µg/g) (Table 1). The percentage relative standard deviation (%RSD) for both retention time and peak area stayed within the established 5% threshold.

**Figure 7:**
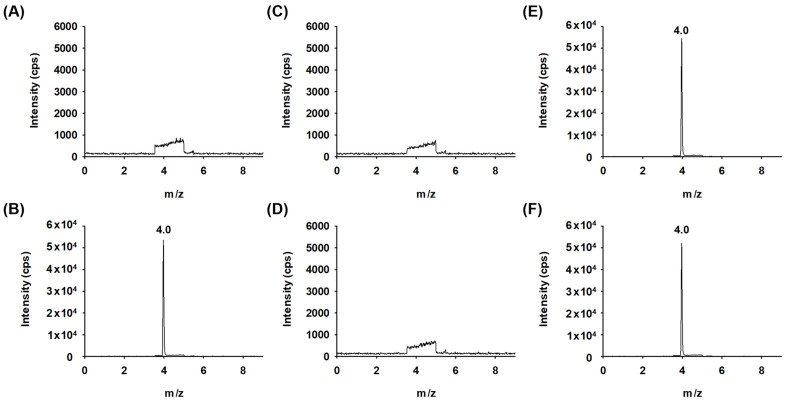
LC-MS/MS chromatograms of DFNB analysis. (**A**) Diluent, (**B**) 50 ng/mL STD solution, (**C**) drug substance sample solution, (**D**) drug product sample solution, (**E**) spiked linezolid API, and (**F**) spiked drug product at 50 ng/mL.

Specificity was tested by analyzing multiple samples, including a blank, a standard solution, an un-spiked sample solution, and a sample solution spiked with DFNB. Across all samples, DFNB consistently showed a retention time of 4.0 min, with no observed interference at its retention time. These findings confirm the specificity of the method, as depicted in Figure 7.

#### 2.3.2. Linearity, LOD, and LOQ

The relationship between signal responses and analyte concentrations was determined using the least squares method over a concentration range of 5–75 ng/mL (corresponding to DFNB in linezolid at 0.83–12.50 µg/g). The coefficient of determination (R^2^) confirmed the linearity of the method, with the mean calibration curve equation being y = 3510*x* − 437 and an R value of 0.9990. A residual plot analysis was performed to assess the fit and validity of the regression model, showing no significant patterns or trends. The limits of detection (LOD) and quantitation (LOQ) were calculated as 1.5 ng/mL and 5.0 ng/mL, respectively, ensuring signal-to-noise (S/N) ratios of at least 3 and 10.

#### 2.3.3. Recovery

In the present study, various solutions with known amounts of DFNB added to both API and drug product were prepared and injected in triplicate. Percent recoveries were calculated, as shown in Table 2. Assays of the linezolid API demonstrated 98.59% overall accuracy with an RSD of 3.86%. Similarly, assays of the drug products yielded 102.15% overall accuracy with an RSD of 2.08%. These results indicate favorable accuracy.

#### 2.3.4. Precision (Repeatability) and Intermediate Precision

Repeatability of the assay was assessed by preparing six replicates of the test solution at 100% of the concentration limit for each sample. The %RSD of retention time for the six replicate preparations did not exceed 1% for both drug substance and drug product, as summarized in Table 3. The assay met the acceptance criteria, requiring a %RSD (n = 6) of no more than 15%.

Intermediate precision was evaluated between two analysts, using the same criteria as for individual repeatability, with the additional requirement of a combined %RSD ≤ 15%. All %RSD values were ≤4.74%, demonstrating that the assay met the acceptance criteria for intermediate precision.

#### 2.3.5. Robustness and Stability

The robustness of the assay was assessed by introducing small variations in column temperature (±4 °C) and flow rate (±0.03 mL/min). Peak area and retention time obtained under these modified conditions were compared with those from the original method parameters. No significant changes were observed, as summarized in Table 4.

Stability experiments were conducted by measuring the percent differences in the standard and sample solutions prepared in diluent and stored in HPLC vials at 10 °C over 18 h. As shown in Table 4, the DFNB concentration remained consistent at the initial time point, after 9 h, and after 18 h, indicating no significant degradation.

#### 2.3.6. Analysis of Drug Samples and Regulatory Considerations

Genotoxic impurities are commonly quantified in parts per million (ppm) relative to the API dose in medicinal products. Linezolid is formulated as oral tablets containing either 300 mg or 600 mg of the API per tablet, with a maximum recommended daily dose of 1200 mg. In this study, a recommended daily intake limit of 10 µg/day for DFNB impurity was established according to the ICH M7 guideline, assuming an exposure duration of 1 to 10 years. To determine the concentration limit for DFNB in ppm, the following calculation was employed:

Limit (ppm) = daily intake of impurity (µg/day)/maximum daily dose of drug (g/day)

This calculation yielded an 8.3 ppm limit. The analytical method developed for this study achieved a quantification limit of 5 ng/mL using mass spectrometry (MS), equivalent to 0.83 ppm in a 6 mg/mL API solution. This value represents 10% of the 8.3 ppm concentration limit for DFNB, as determined based on the threshold of toxicological concern (TTC). Recovery of the analyte at the LOQ was within the acceptable range of 99% to 103%, with a %RSD between 2.36% and 3.54%. These findings confirm the precision and accuracy of this method for quantifying DFNB in linezolid APIs and drug products.

This method was applied to evaluate DFNB contamination in three batches of linezolid APIs and drug products available in the local pharmaceutical market. Each product underwent triplicate analysis, and no DFNB was detected in any of the samples.

## 3. Materials and Methods

### 3.1. Materials and Reagents

3,4-Difluoronitrobenzene (DFNB), linezolid active pharmaceutical ingredient (API), acetonitrile, and methanol were sourced from Sigma-Aldrich (St. Louis, MO, USA). Ultrapure water was provided by J.T. Baker (Phillipsburg, NJ, USA). Formic acid and ammonium formate used as eluent additives were obtained from Thermo Fisher Scientific (Waltham, MA, USA). All materials used were of HPLC-grade purity. Linezolid drug products, including tablets, were supplied by a local pharmaceutical manufacturer.

### 3.2. HPLC Conditions

Chromatographic analysis was performed using a Nexera X3 system (Shimadzu, Kyoto, Japan), which included components such as a controller, autosampler, column oven, solvent delivery module, and a photodiode array detector. An Ascentis^®^ Express C18 column (2.1 × 100 mm, 2.7 µm, Supelco, Bellefonte, PA, USA) was utilized, maintained at 40 °C in the column oven. The autosampler cooler was set to 10 °C, and a 3 µL injection volume was used for LC-MS/MS analysis. The mobile phases consisted of water (Phase A) and methanol (Phase B), with a gradient flow at 0.3 mL/min programmed as follows: 0–2 min, 40% (B); 2–4 min, 40–100% (B); 4–6 min, 100% (B); 6–6.1 min, 100–40% (B); and 6.1–9 min, 40% (B). A photodiode array detector scanned wavelengths from 190 to 400 nm for analyte detection.

### 3.3. Mass Spectrometer Conditions

The HPLC system was coupled to an AB Sciex QTrap 5500 Plus mass spectrometer (Framingham, MA, USA) optimized for LC-MS/MS operation. Negative ionization was performed using atmospheric pressure chemical ionization (APCI), and analytes were monitored at ion transitions of *m*/*z* 156 → 126 for DFNB. The operating parameters of mass spectrometer included a source temperature of 500 °C, nebulizer current (NC) of −5 µA, curtain gas (CUR) at 35 psi, nebulizer gas (GS1) at 55 psi, and collision gas (CAD) at 9. High-purity nitrogen and zero-grade air were provided by a gas generator (Euro Science Co., Ltd., Seongnam-si, Gyeonggi-do, Republic of Korea). DFNB-specific settings included a declustering potential (DP) of −35 V, collision energy (CE) of −25 V, and collision cell exit potential (CXP) of −12 V. To prevent contamination of the ion source, the eluent was diverted to waste through a switching valve for the first minutes. Data acquisition and processing were carried out using Analyst 1.7.3 software from AB Sciex.

### 3.4. Standard Solution Preparation

A stock solution of DFNB was prepared in methanol at a concentration of 1 mg/mL and then diluted with methanol to a concentration of 1000 ng/mL. The resulting DFNB concentrations for calibration were 5, 15, 25, 50, 60, and 75 ng/mL. The 100% standard solution, corresponding to the concentration limit, was 50 ng/mL. Recovery test solutions were prepared by spiking standard solutions into drug substance and drug product matrices, with subsequent sample preparation identical to that used for the analytical samples, yielding DFNB solutions at 5, 25, 50, and 75 ng/mL.

### 3.5. Sample Preparation

#### 3.5.1. Drug Substance

A 60 mg sample of linezolid API was added to a disposable 15 mL glass centrifuge tube and dissolved in 10 mL of methanol. The solution was vortexed for 1 min and then centrifuged at 4000 rpm for 10 min. The supernatant was filtered using a 0.22 µm PVDF syringe filter, discarding the first 1 mL. Finally, 1 mL of the filtered solution was transferred to an autosampler vial for LC-MS/MS analysis.

#### 3.5.2. Drug Product

Tablet powders equivalent to 60 mg of linezolid API were weighed and transferred into a disposable 15 mL glass centrifuge tube. Then, 10 mL of methanol was added, and the mixture was vortexed for approximately 1 min. The subsequent preparation steps were identical to those used for the drug substance.

### 3.6. Method Validation

The method for determining DFNB in linezolid was validated according to ICH Q2 (R2) guidelines [35], evaluating parameters such as system suitability, specificity, linearity, limits of detection (LOD) and quantitation (LOQ), precision, accuracy, repeatability, intermediate precision, robustness, and solution stability.

System suitability was assessed by analyzing the variability of peak area and retention time across six injections, ensuring a %RSD of ≤5%. Specificity was confirmed by injecting a blank solution (methanol), standard solution, drug substance and drug product sample solutions, and a sample spiked with DFNB, ensuring no interference at retention time of the analyte. Linearity was evaluated using six concentrations ranging from 5 to 75 ng/mL, corresponding to 10–150% of the specified DFNB limit, with three replicates at each level. A residual plot analysis confirmed the linearity of the method with R values exceeding 0.990. LOD and LOQ were established at levels, respectively, based on signal-to-noise ratios (S/N) of ≥3 and ≥10. Accuracy was assessed at four concentrations (5, 25, 50, and 75 ng/mL) within the calibration range with triplicate analyses. Mean assay values, standard deviations, %RSD, and percent recovery were calculated for each sample. Repeatability was assessed by conducting six measurements at the 100% test concentration, with the results expressed as %RSD, and an acceptance criterion of ≤15%. Intermediate precision was assessed by two analysts, each following the same criteria for individual repeatability, along with an additional requirement that the combined %RSD for both analysts be ≤15%. Robustness was evaluated by varying the eluent flow rate and column temperature by ±10%, with %RSD calculated for peak response and retention time. Solution stability was assessed by determining the %difference in DFNB peak area in a spiked sample solution over 18 h at 10 °C.

## 4. Conclusions

This study presents a novel, quantitative, rapid, and highly reliable LC-APCI-MS/MS method for the analysis of 3,4-difluoronitrobenzene (DFNB) residues in linezolid. Under negative APCI conditions, DFNB generates characteristic ion formations, such as [M]^•−^ through electron capture and [M − F + O]^−^ via substitution reactions. Fragment ions corresponding to the loss of NO and NO_2_ from the parent ion were consistently and reliably observed. The unique features of this method, including carefully optimized MRM using MS/MS ion transitions of the substitution product, ensure accurate detection of this specific impurity. This method stands out due to its precise and reliable performance, which allows for the detection and accurate measurement of DFNB at very low concentrations, down to 5 ng/mL (0.83 µg/g). Additionally, validation of this method, performed according to ICH Q2(R2) guidelines, demonstrated excellent precision, reproducibility, selectivity, sensitivity, and linearity, making it highly suitable for routine quality control in pharmaceutical manufacturing. The effectiveness of this LC-MS/MS method was successfully demonstrated through its practical application in analyzing DFNB impurities in linezolid drug formulations, providing critical data to support comprehensive quality assessments of pharmaceutical products. This method fills a crucial gap in current drug quality control practices, offering a reliable and efficient solution for the detection of DFNB, a key impurity in pharmaceutical formulations. Therefore, this innovative LC-MS/MS technique represents a significant advancement in impurity analysis, ensuring high accuracy and efficiency in pharmaceutical quality assessment.

## Figures and Tables

**Table 1:** Results of system suitability.

Parameter		Value
Retention time (RT, min)		4.0
Theoretical plate number (N)		36,594
HETP (mm)		0.0027
Capacity factor (*k’*)		5.67
Symmetry factor		1.23
System precision (%RSD, N = 6)	Area	1.17
	Retention time	0.13

**Table 2:** Accuracy for DFNB in API and drug product.

Nominal Conc. (ng/mL)	Drug Substance (n = 3)			Drug Product (n = 3)		
	Measured Conc. (ng/mL, Mean ± SD)	Recovery (%)	RSD %	Measured Conc. (ng/mL, Mean ± SD)	Recovery (%)	RSD %
5	5.14 ± 0.18	102.78	3.54	5.20 ± 0.48	99.71	2.36
25	24.31 ± 0.78	97.24	3.22	25.59 ± 0.46	102.38	1.78
50	47.54 ± 1.02	95.08	2.15	51.92 ± 0.50	103.84	0.97
75	74.43 ± 1.50	99.24	2.01	77.01 ± 0.80	102.68	1.04
Overall	-	98.59	3.86	-	102.15	2.08

**Table 3:** Repeatability and intermediate precision.

Matrix	Analyst	Retention Time (min)			Calculated Conc. (ng/mL)		
		Mean	SD	%RSD	Mean	SD	%RSD
drug substance	Analyst 1, day 1 (n = 6)	4.0	0.01	0.10	48.70	0.93	1.91
	Analyst 2, day 2 (n = 6)	4.0	0.00	0.00	48.94	2.22	4.53
	Combined (n = 12)	4.0	0.00	0.08	48.82	1.63	3.33
drug product	Analyst 1, day 1 (n = 6)	4.0	0.01	0.11	51.56	0.91	1.76
	Analyst 2, day 2 (n = 6)	4.0	0.00	0.00	49.44	3.01	6.09
	Combined (n = 12)	4.0	0.00	0.09	50.50	2.39	4.74

**Table 4:** Robustness and stability.

Robustness (%RSD, n = 3)				Solution Stability (% Difference, n = 3)					
Column Temp. (40 ± 4 °C)		Flow Rate (0.3 ± 0.03 mL/min)		Standard Solution		In Drug Substance		In Drug Product	
Area	RT	Area	RT	9 h	18 h	9 h	18 h	9 h	18 h
2.60	1.49	4.00	3.59	−6.44	−2.97	−5.76	6.87	0.51	5.17

## Data Availability

All data are included in the article.
